# The Super-Seniors Study: Phenotypic characterization of a healthy 85+ population

**DOI:** 10.1371/journal.pone.0197578

**Published:** 2018-05-24

**Authors:** Julius Halaschek-Wiener, Lauren C. Tindale, Jennifer A. Collins, Stephen Leach, Bruce McManus, Kenneth Madden, Graydon Meneilly, Nhu D. Le, Joseph M. Connors, Angela R. Brooks-Wilson

**Affiliations:** 1 Canada’s Michael Smith Genome Sciences Centre, British Columbia Cancer Agency (BCCA), Vancouver, British Columbia, Canada; 2 Biomedical Physiology and Kinesiology, Simon Fraser University, Burnaby, British Columbia, Canada; 3 PROOF Centre of Excellence, University of British Columbia, Providence Health Care, Vancouver, British Columbia, Canada; 4 Faculty of Medicine, University of British Columbia, Vancouver, British Columbia, Canada; 5 Cancer Control Research, BCCA, Vancouver, British Columbia, Canada; 6 Centre for Lymphoid Cancer, BCCA, Vancouver, British Columbia, Canada; University of Palermo, ITALY

## Abstract

**Background:**

To understand why some people live to advanced age in good health and others do not, it is important to study not only disease, but also long-term good health. The Super-Seniors Study aims to identify factors associated with healthy aging.

**Methods:**

480 healthy oldest-old ‘Super-Seniors’ aged 85 to 105 years and never diagnosed with cancer, cardiovascular disease, diabetes, dementia, or major pulmonary disease, were compared to 545 mid-life controls aged 41–54, who represent a group that is unselected for survival from late-life diseases. Health and lifestyle information, personal and family medical history, and blood samples were collected from all participants. Super-Seniors also underwent four geriatric tests.

**Results:**

Super-Seniors showed high cognitive (Mini-Mental State Exam mean = 28.3) and functional capacity (Instrumental Activities of Daily Living Scale mean = 21.4), as well as high physical function (Timed Up and Go mean = 12.3 seconds) and low levels of depression (Geriatric Depression Scale mean = 1.5). Super-Seniors were less likely to be current smokers than controls, but the frequency of drinking alcohol was the same in both groups. Super-Seniors were more likely to have 4 or more offspring; controls were more likely to have no children. Female Super-Seniors had a mean age of last fertility 1.9 years older than controls, and were 2.3 times more likely to have had a child at ≥ 40 years. The parents of Super-Seniors had mean ages of deaths of 79.3 years for mothers, and 74.5 years for fathers, each exceeding the life expectancy for their era by a decade.

**Conclusions:**

Super-Seniors are cognitively and physically high functioning individuals who have evaded major age-related chronic diseases into old age, representing the approximately top 1% for healthspan. The familiality of long lifespan of the parents of Super-Seniors supports the hypothesis that heritable factors contribute to this desirable phenotype.

## Introduction

Healthy aging and extreme longevity are phenotypes that many hope to achieve. For many, however, old age is accompanied by poor health. Longevity refers to the length of time an individual lives, their lifespan, whereas healthy aging refers to a person’s ‘health span’. The majority of longevity can be attributed to environmental and lifestyle factors; however, 15–30% of adult lifespan is heritable [1]. While the heritability of longevity is minimal before age 60, it increases at more advanced ages [2]. Some longevity genes have been identified in model organisms; however, few findings have been replicable in humans with the exceptions of *APOE* and *FOXO3*, reviewed in [3].

According to the US Centre for Disease Control, the leading causes of death over the age of 65, in descending order, are: diseases of the heart, malignant neoplasms, chronic lower respiratory diseases, cerebrovascular disease, Alzheimer disease (AD), and diabetes mellitus [4]. We describe here the ascertainment and characterization of 480 oldest old who have never been diagnosed with any of these diseases.

Several research studies have collected long-lived individuals [5–10], most with the goal of studying longevity or exceptional longevity. Recent insights have also been gained from large cross-sectional population studies [11, 12]. The Super-Seniors Study examines individuals over 85 with a well-characterized health phenotype free of five specific major age-related diseases, in order to study healthy aging. This focus is shared by the Wellderly study [13], which researches healthy elderly aged 80 and over.

We have collected a group of oldest-old “Super-Seniors”, aged 85 years and older who have never been diagnosed with cancer, cardiovascular disease (CVD), diabetes, dementia or major pulmonary disease; as well as a group of mid-life controls recruited randomly with respect to health. The Super-Seniors represent a group of individuals who have not only survived to at least 85, but have done so free of the major chronic diseases that lead to decreased quality of life and early death. The Super-Senior phenotype is also economically relevant—the diseases they have evaded are among the most common and therefore expensive to provide care for within a healthcare system. Because most individuals in developed countries live to at least age 50, the mid-life group represents a set of individuals who are as yet unselected for the age-related diseases that the Super-Seniors have avoided.

To some, 85 years may now seem too young to be considered long-lived [14]. While individuals born more recently have tended to live longer, for those born in the years that the Super-Seniors were, reaching 85 years was quite a feat (S1 Fig).

Future work will compare the Super-Seniors to population-based mid-life controls as a strategy to identify genetic factors that may contribute to their long-term good health. Here we characterize the health, family history and lifestyle of the Super-Seniors.

## Methods

### Recruitment

This study was approved by the University of British Columbia (BC)-BC Cancer Agency Research Ethics Board and the Research Ethics Board of Simon Fraser University. All participants gave written informed consent.

Eligibility criteria for being a Super-Senior included: being 85 years or older and self-reporting as never having had cancer (except non-melanoma skin cancer), CVD, diabetes, dementia, or major pulmonary disease (except asthma). Controls were aged 41–54 years and not selected for health.

Using lists from the BC Ministry of Health Medical Services Plan (MSP), which includes 98% of BC residents, we contacted individuals living in Metro Vancouver, BC, Canada 85 or older, or 40–50 years old. Additional invitations were sent to potential controls accompanied by an offer of a $50 honorarium. Super-Seniors were also identified by BC Stats, which had confidential access to the Insurance Corporation of BC drivers license database and could allow contact of currently licensed drivers, or were those who volunteered following press coverage. In an attempt to collect additional Super-Seniors of Asian ancestry, advertisements were placed in Chinese-language newspapers. Finally, to collect a greater number of European controls, later mail-outs included only midlife individuals with non-Asian surnames.

After initial contact by mail, potential Super-Seniors were screened by phone to determine eligibility. After receiving written informed consent, a home or telephone interview was arranged for Super-Seniors and controls, respectively. Ascertainment and sample collection took place between 2004 and 2007, resulting in the shifting age range from the intended 40–50 to 41–54.

### Data collection

Super-Seniors were visited at home by an interviewer and asked for personal and family medical history, to show all prescription and non-prescription medications, and to perform geriatric tests. Test scores did not affect eligibility.

Controls were asked the same personal/family medical history questions, but were not selected for health or disease status.

Ethnicity of the four grandparents was collected, and a composite ethnicity was determined for each participant. A participant’s ethnicity was categorized as unknown if they did not know the ethnicity of all four grandparents. If participants were unsure about the age of death of their parents, an approximation was made; for example, ‘mid-sixties’ was approximated as 65 years.

30mL of non-fasting blood was drawn from each participant. Super-Seniors were visited by a phlebotomist; controls visited a commercial clinical laboratory. DNA was extracted using the PureGene DNA isolation kit (Gentra Systems, MN).

### Phenotypic review

Health data of the Super-Seniors were reviewed and potential cases were excluded based on presence of disease but not based on intermediate phenotypes such as high blood pressure. Medications were reviewed and potential Super-Seniors excluded if they were taking a drug used exclusively to treat cancer, CVD, dementia, pulmonary disease or diabetes. Participants with borderline health status (generally those with an asymptomatic arrhythmia or chronic bronchitis) were excluded from subsequent analyses.

### Statistical analysis

Statistical tests were run in JMP Version 11 [15].

## Results

### Recruitment

**Fig 1:**
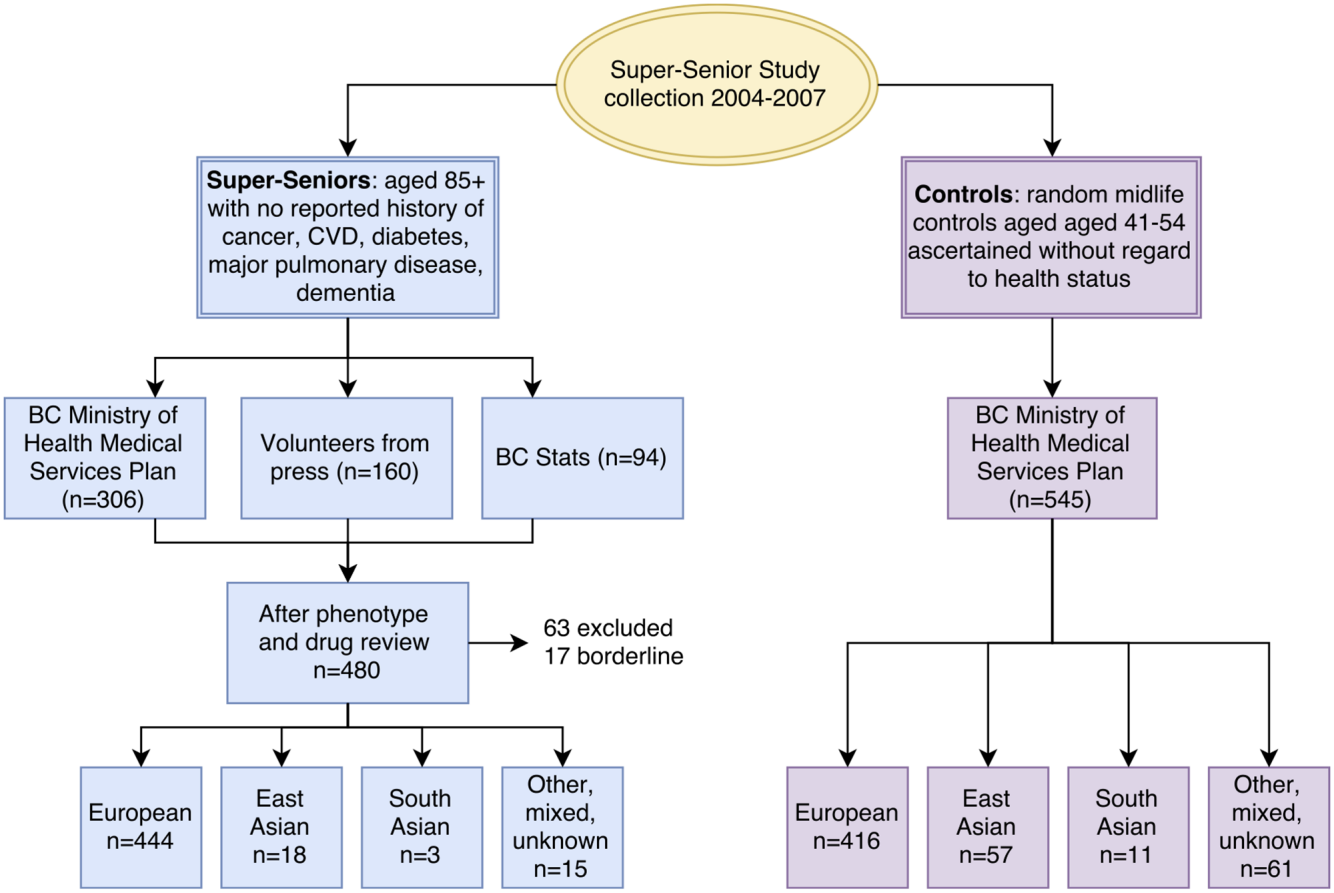
Recruitment of Super-Seniors and controls.

Recruitment is summarized in Fig 1 and S2 Fig. An initial mailing was sent to BC MSP subscribers: 8415 individuals aged 85 or more, and 3920 aged 40–50. Of potential Super-Seniors, 4261 (50.6%) had incorrect contact information, were unable to be contacted, or did not speak English; 628 (7.5%) were deceased; 1059 (12.6%) refused without determining eligibility; 2161 (25.7%) were not eligible; and 306 (3.6%) participated. Of the potential controls, 2884 (73.6%) had incorrect contact information, were unable to be contacted, or did not speak English; 491 (12.5%) refused, and 545 (13.9%) participated. 12.4% of potential Super-Seniors who were interested were eligible. The consent rate for controls was 52.6% (S2 Fig).

In addition to those identified through BC MSP, 160 Super-Seniors volunteered and 94 were identified by BC Stats. After review of interview data and medications, 63 individuals were excluded and 17 were borderline, resulting in 480 Super-Seniors and 545 controls.

### Descriptive statistics

There were 325 female and 155 male (32.3%) Super-Seniors and 336 female and 209 male (38.3%) controls (Table 1 and S3 Fig). Super-Seniors were aged 85–105 (mean 88.5 years); controls were aged 41–54 (mean 46.7 years). 92.5% of Super-Seniors and 76.3% of controls were of European ancestry (Fig 1 and S1 Table). Super-Seniors had a mean BMI of 25.7 (SD 4.7), while controls had a mean BMI of 24.5 (SD 3.9).

**Table 1:** Characteristics of the study participants.

		Super-Seniors			Controls		
		Male	Female	Total	Male	Female	Total
**Descriptive statistics**	N	155	325	480	209	336	545
	Age—mean (SD)	88.7 (2.9)	88.5 (2.9)	88.5 (2.9)	46.8 (3.2)	46.6 (3.4)	46.7 (3.3)
	Range (years)	85–100	85–105	85–105	41–53	41–54	41–54
	Birth year—mean	1916	1916	1916	1958	1958	1958
	BMI—mean (SD)	25.0 (3.4)	24.3 (4.1)	24.5 (3.9)	26.7 (4.0)	25.0 (5.0)	25.7 (4.7)
	Range (kg/m^2^)	19.0–42.5	15.1–42.1	15.1–42.5	18.4–46.8	16.8–48.4	16.8–48.4
**Smoking**	Smoker—current (%)	4 (2.6)	3 (0.9)	7 (1.5)	25 (12.0)	33 (9.8)	58 (10.6)
	Smoker—never (%)	52 (33.5)	189 (58.2)	241 (50.2)	107 (51.2)	161 (47.9)	268 (49.2)
	Smoker—quit (%)	99 (63.9)	133 (40.9)	232 (48.3)	77 (36.8)	142(42.3)	219 (40.2)
	Years smoked (among quitters)—mean (SD)	31.9 (17.4)	27.8 (17.8)	29.4 (17.8)	14.5 (10.2)	13.5 (8.6)	13.8 (9.2)
	Pack years smoked (among quitters)—mean (SD)	24.7 (28.2)	15.4 (20.8)	19.3 (24.6)	10.3 (10.9)	9.1 (9.4)	9.6 (9.9)
**Activity**	Activity—none (%)	25 (16)	71 (22)	96 (20)	51 (24)	81 (24)	132 (24)
	Activity—walking (%)	54 (35)	95 (29)	149 (31)	24 (11)	66 (20)	90 (17)
	Activity—exercise (%)	75 (49)	158 (49)	233 (49)	134 (64)	186 (56)	320 (59)
**Alcohol**	Alcohol—beer* (%)	28 (6)	24 (5)	52 (11)	130 (24)	75 (14)	205 (38)
	Alcohol—spirits* (%)	54 (11)	80 (17)	134 (28)	60 (11)	76 (14)	136 (25)
	Alcohol—wine* (%)	76 (16)	180 (38)	256 (53)	125 (23)	225 (41)	350 (64)
	Alcohol—none (%)	32 (7)	91 (19)	123 (26)	29 (5)	59 (11)	88(16)
**Fertility**	Number of offspring	2.6 (1.6)	2.5 (1.7)	2.6 (1.7)	1.5 (1.2)	1.7 (1.2)	1.6 (1.2)
	Range	0–12	0–8	0–12	0–5	0–5	0–5
	Had offspring	131	284	415	139	248	387
	Age of last fertility—mean (SD)	35.8 (6.3)	33.7 (5.8)	-	34.1 (5.6)	31.8 (5.1)	-
	Range (years)	23–54	19–47	-	20–47	17–45	-
	Had offspring 40+ years	34	47	-	25	20	-
	Age of 40+ parents—mean (SD)	44.1 (3.4)	42.6 (2.2)	-	42.4 (1.9)	41.3 (1.6)	-
	Had offspring 35+ years	68	128	-	63	75	-
	Age of 35+ parents—mean (SD)	40.7 (4.3)	38.8 (3.4)	-	39.2 (3.0)	37.9 (2.4)	-
**Parents**	Maternal age of death	79.6 (15.6)	79.1 (15.6)	79.3 (15.6)	-	-	-
	Paternal age of death	75.0 (15.9)	74.2 (16.1)	74.5 (16.0)	-	-	-
**Geriatric tests**	TUG—mean (SD)	12.2 (4.0)	12.3 (4.5)	12.3 (4.3)	-	-	-
	MMSE—mean (SD)	28.1 (1.7)	28.4 (1.7)	28.3 (1.7)	-	-	-
	GDS—mean (SD)	1.5 (1.8)	1.6 (1.8)	1.5 (1.8)	-	-	-
	IADL—mean (SD)	21.7 (3.0)	21.2 (3.7)	21.4 (3.5)	-	-	-

*Categories are not mutually exclusive

### Functional tests

**Fig 2:**
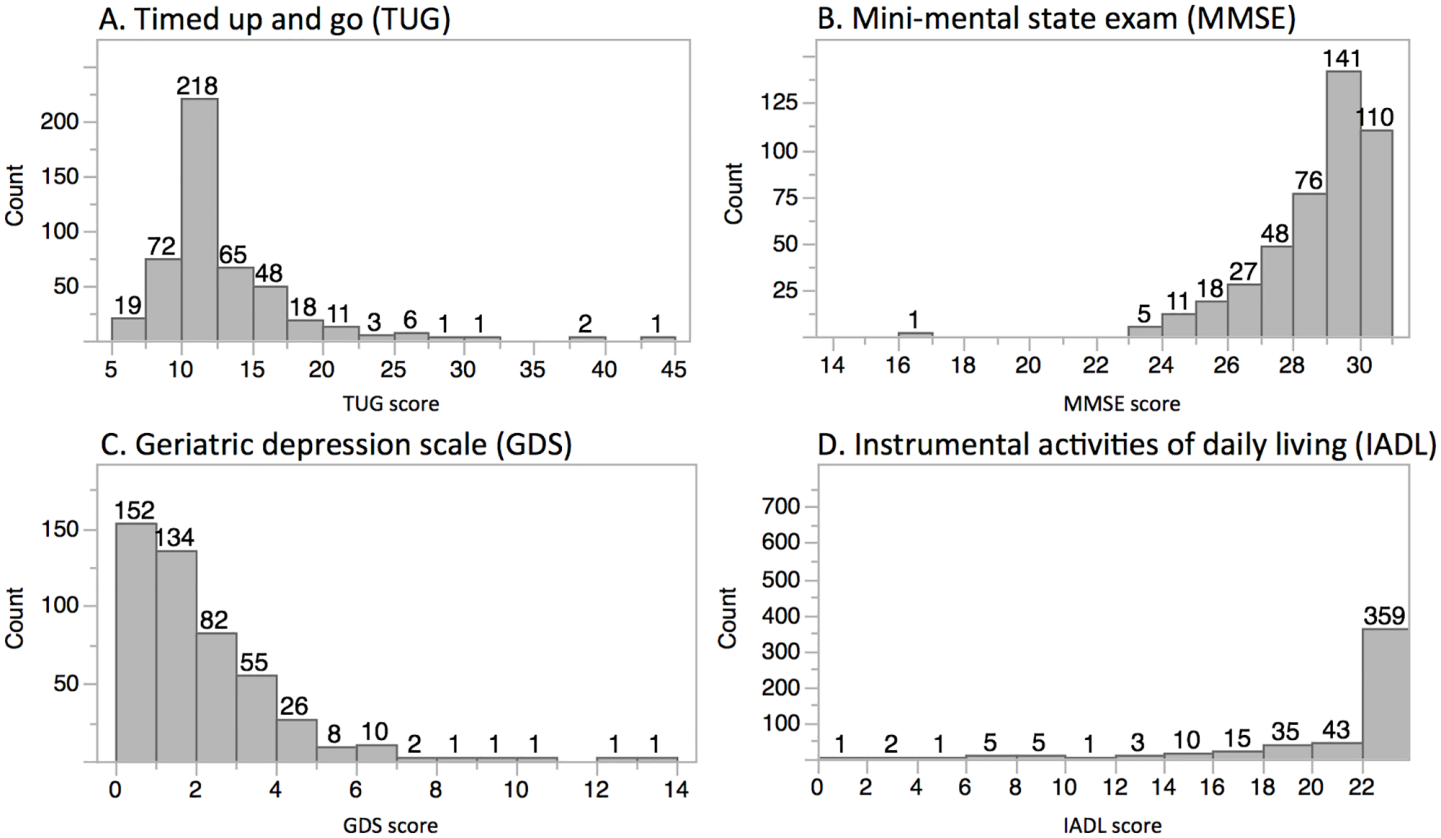
Distribution of functional test scores of the Super-Seniors.

Super-Seniors scored high on the Mini-Mental State Exam (MMSE) [16], mean = 28.3 (SD 1.7) and Instrumental Activities of Daily Living Scale (IADL) [17], mean = 21.4 (SD 3.5), and low on the Timed Up and Go test (TUG) [18], mean = 12.3 seconds (SD 4.3) and Geriatric Depression Scale (GDS) [19], mean = 1.5 (SD 1.8) (Fig 2). Inability to complete the MMSE as a result of vision deficits resulted in 35 scores being excluded. The TUG was not administered if the participant was not able to ambulate independently. Seven MMSE, 15 TUG, and 6 GSD scores were missing. No differences were detected between the scores for men and women for any test.

### Lifestyle

Smoking status was divided into current, never, and quit (Table 1). Controls were 8.0 fold more likely to be current smokers than cases (95%CI = 3.36–15.8, p<0.0001)(S2 Table). There was a difference in proportion between male and female never smokers and quitters in Super-Seniors (X^2^ = 24.2, p<0.0001): female Super-Seniors were more likely to be never smokers (58.7%) than men (34.4%), and men were more likely to be quitters (65.6%) than women (41.3%). No difference in smoking status was detected between sexes in controls.

Among quitters, Super-Seniors smoked a mean of 29.4 years (SD = 17.8) and 19.3 pack years (SD = 24.6) (pack year = packs smoked/day*years smoked); whereas controls who quit smoked a mean of 13.8 years (SD = 9.2) and 9.6 pack years (SD = 9.9). Although there was no difference in the mean number of years that male and female Super-Seniors smoked, male Super-Seniors smoked more heavily, for a mean of 9.32 pack years more than females (SE = 3.4, 95%CI = 2.6–16.0, p = 0.007). Super-Senior quitters started smoking at a mean age of 20.5 years (SD = 8.1) and quit at a mean age of 50.0 years (SD = 18.2); control quitters started smoking at a mean age of 16.6 years (SD = 3.3) and quit at a mean age of 30.5 years (SD = 8.8).

49% of Super-Seniors and 59% of controls reported exercising (X^2^ = 10.44, p<0.0012), with controls more likely to engage in exercise other than walking. No significant difference was observed in the proportion of Super-Seniors and controls who drank alcohol.

#### Number of offspring and age of fertility

There was a difference in the proportion of number of offspring between Super-Seniors and controls (X^2^ = 107.0, p<0.0001)(S3 Table). Super-Seniors had a mean number of offspring (2.6, SD = 1.7) that was higher than controls (mean = 1.6, SD = 1.2);

284 Super-Senior and 248 control females gave birth. Super-Senior women had a mean age of last fertility 1.9-years older than control women (SE = 0.5, 95%CI = -2.8– -1.9, p<0.0001). There was no evidence of a difference in mean age of first fertility. Super-Senior men also had their last child a mean of 1.7 years older than control men (SE = 07, 95%CI = -0.2– -3.1, p = 0.011).

47 (16.5%) Super-Seniors and 20 (8.1%) controls who reproduced gave birth at ≥40 years, at a mean age of 42.6 (SD = 2.2) and 41.3 (SD = 1.6) years, respectively. 128 (45.1%) Super-Seniors and 75 (30.2%) controls gave birth ≥35, at a mean age of 38.8 (SD = 3.4) and 37.9 (SD = 2.4). Among women who gave birth, Super-Seniors were 2.3 times more likely to have had a child at ≥40 years (95%CI = 1.3–3.9, p = 0.004), and 1.9 times more likely to have had a child at ≥35 years (95%CI = 1.3–2.7).

#### The parents of Super-Seniors lived longer than their contemporaries

The Super-Seniors reported their parents’ age at death to be between 21–110 years for their mothers (mean = 79.3, SD = 15.6), and 26–102 years for their fathers (mean = 74.5, SD = 16.0). The control parents’ ages of death ranged from 26 years to still alive for mothers, and 29 to still alive for fathers.

The parents of the Super-Seniors were born between ~1880–1905. The earliest survival statistics for North America are Americans born in 1900 [4]. We compared age at death of the parents of Super-Seniors (who we know lived to reproductive age) to individuals born in 1900 who survived to age 21. 50% of 21 year olds born in 1900 lived to 67 years (66 for men, 68 for women). The mothers and fathers of the Super-Seniors therefore lived 11.3 and 8.5 years (average of 9.9 years) longer than the 1900 birth cohort.

## Discussion

We have established a collection of healthy oldest-old and a mid-life control group recruited from population-based lists. Here, we describe the characteristics and cognitive and physical function of the Super-Seniors. We also document major lifestyle factors such as smoking and alcohol consumption to allow adjustment for these factors in future genetic analyses.

Hidden differences in ethnicities in case-control studies can lead to false positive genetic findings. Early in recruitment, we noted a difference in ethnicity between the Super-Seniors and controls. Over time, Metro Vancouver has seen increasing immigration by non-European groups. We attempted to equalize the composition of the two groups by identifying Asian-ancestry Super-Seniors, with little success, so instead over-collected controls of European ancestry.

We define the Super-Senior phenotype as oldest-old (≥85 years) who have never been diagnosed with any of five major diseases that are the leading causes of death over the age of 65 [4]. 12.4% of seniors over age 85 who were contactable and interested were eligible. Given that 28.5% of Canadians age 85 or older have dementia [20], the eligibility rate of living individuals is therefore closer to 8.9%. Furthermore, only 9.0% of individuals born in 1916 lived to be 85 [21]. The proportion of the 1916 birth cohort who went on to become Super-Seniors is therefore approximately 0.80%, making Super-Senior status the ‘top 1%’ elite health and survival phenotype.

Super-Seniors had a mean TUG of 12.3 seconds, indicating that the majority are able to ambulate independently [18]. The Newcastle 85+ Study observed a baseline TUG of 18.6 ± 14.7 (n = 735) and a 5-year follow-up TUG of 20.7 ± 12.0 (n = 271) [22]. A study of community-dwelling Taiwanese individuals 65+ years of age found that physical fitness indicators, including the TUG, are associated with successful aging [23].

The median MMSE score of the Super-Seniors was 29, with a single participant scoring <19. In the Leiden Longevity Study, [10] (men 89+, women 91+) the median MMSE score was 25 and 14% scored <19. In the Taiwanese study only 73.5% had an MMSE ≥24 [23]. By selecting for a disease-free state that excluded dementia, the Super-Seniors are a cognitively high functioning group, even when compared to other long-lived groups.

A GDS score ≥ 5 has been found to be a sensitive and specific cutoff for depression [24]. The Super-Seniors had a mean GDS score of 1.5. The IADL assesses performance of daily tasks [17]. Although the mean IADL score of Super-Seniors was high, there were a few low scores, with the three lowest belonging to individuals who used wheelchairs. Because a minority of participants lived with family or in an assisted living community, lower IADL scores in some instances reflected their responsibilities rather than their abilities.

Several differences between the Super-Seniors and controls are expected and reflect population trends over time, including smoking habits and family size. Super-Senior women were less likely to be smokers than Super-Senior men, but there was no difference between male and female smoking rates in the controls. This is consistent with Canadian smoking trends [25]. Controls were more likely to be current smokers; one reason for this is that some smokers in the Super-Senior age range would likely have developed smoking-related diseases that would make them ineligible. There was no evidence of a difference in the proportion of Super-Seniors and controls who drank alcohol.

There were higher proportions of controls having no offspring and Super-Seniors having 4+ offspring. This likely reflects changes in family size over time; however, some controls in their early 40s could go on to have additional offspring.

Super-Senior women had an older mean age of last fertility and were 2.3 times more likely to give birth at ≥40 years, compared to controls. Associations have been found between late childbirth (40+) and increased survival in women [26–28]. Female centenarians were four times more likely to have had children in their forties than a group from the same birth year who died at age 73 [29]. Late fertility, and more specifically the ability to bear a child after age 40, may be a sign of slower biological aging [29]. On average, Super-Senior fathers had their last child at an older age than control fathers, suggesting that in some cases the advanced maternal age of the Super-Senior women may have been because they had to wait until after the war to start their families.

Super-Senior parents lived substantially longer than their contemporaries, suggesting a familial tendency towards long life. The true difference in lifespan is probably greater, as many of the parents of the Super-Seniors were likely born before 1900, when life expectancy was even lower.

The mid-life control group is intended for use only for genetic comparisons with the Super-Seniors, not for epigenetics or formal comparison of lifestyle or other non-genetic factors. The latter quantities cannot be compared between these groups because they have lived in different eras and would be expected to show potentially confounding cohort effects.

The ideal control group for the Super-Seniors would be individuals from the same birth cohort who did not successfully achieve the Super-Senior phenotype. Clearly it is not feasible to obtain DNA from such a control group. Instead, we use the strategy of comparing the elite Super-Seniors to a group that has not been selected for survival in later life. From the survival curves in S1B Fig, in 1958 (the mean birth year of the controls), the curve is nearly flat until approximately age 50 because relatively few people born in 1958 died before that age; they were therefore largely unselected for survival up to that point.

If we were to compare Super-Seniors to age-matched individuals, we would be comparing healthy oldest old to unhealthy oldest old. Such individuals would have in common the fact that they all survived to at least age 85, and the phenotypic (and presumably genotypic) differences between them would be more subtle, and harder to detect, than those that could differ between the highly selected Super-Seniors vs. the largely unselected mid-life individuals. Importantly, an age-matched control group does not allow us to study genetic factors that affect survival to the oldest-old age category, or those genetic factors that might influence both survival and health late in life.

A limitation of our study design is that the midlife group is expected to contain a small minority of people (approximately 1%) who would have been destined to become Super-Seniors, had they been born circa 1916 (we note that being born in 1916 and surviving to 85, or being born in 1958 and surviving to 85, are not the same survival phenotype because the threats to survival and health differ between the two eras). The presence of such rare individuals amongst the midlife controls slightly reduces the statistical power of the study. Another limitation is that small sample size limits the statistical power of the current sample set. To overcome this limitation a Phase 2 recruitment is in progress; we also intend to combine the data from the Super-Seniors Study with aging consortia for meta-analyses once we have completed initial genetic analyses. We plan a future recruitment of age-matched less healthy elderly to use as another comparison population.

We have established and characterized an initial cohort in which to study the genetics of healthy aging [30–33], with Super-Seniors representing an elite group in terms of healthspan. The study that is most comparable to the Super-Seniors is the Wellderly Study of healthy elderly aged 80 and older [13]. While healthy aging is defined similarly between these two studies, the Super-Seniors are on average 4.3 years older (average 88.5 years vs. 84.2 years) and have a higher proportion of women (67.7% vs. 60.7%). Geriatric test scores demonstrate that Super-Seniors are a cognitively and physically high functioning group in addition to being healthy oldest old. The long lifespan of the parents of Super-seniors supports the hypothesis that heritable factors contribute to this desirable phenotype.

## Supporting information

S1 Fig Life expectancy in Canada. (PDF)

S2 Fig Collection of Super-Seniors and controls from BC Medical Services Plan lists. (PDF)

S3 Fig Age distribution of participants in the Super-Seniors Study. (PDF)

S1 Table Distribution of ethnicity in Super-Seniors and controls. (PDF)

S2 Table Contingency table of smoking status in Super-Seniors and controls. (PDF)

S3 Table Contingency table of number of offspring in Super-Seniors and controls. (PDF)

S4 Table Super-Senior study phenotype data. (XLSX)
