# Supplementary material for: The Super-Seniors Study: Phenotypic characterization of a healthy 85+ population
Source: PLoS One. 2018 May 24;13(5):e0197578. doi: 10.1371/journal.pone.0197578 (PMC5967696; doi:10.1371/journal.pone.0197578)
Supplement: S1 Fig — (PDF) [file pone.0197578.s001.pdf]

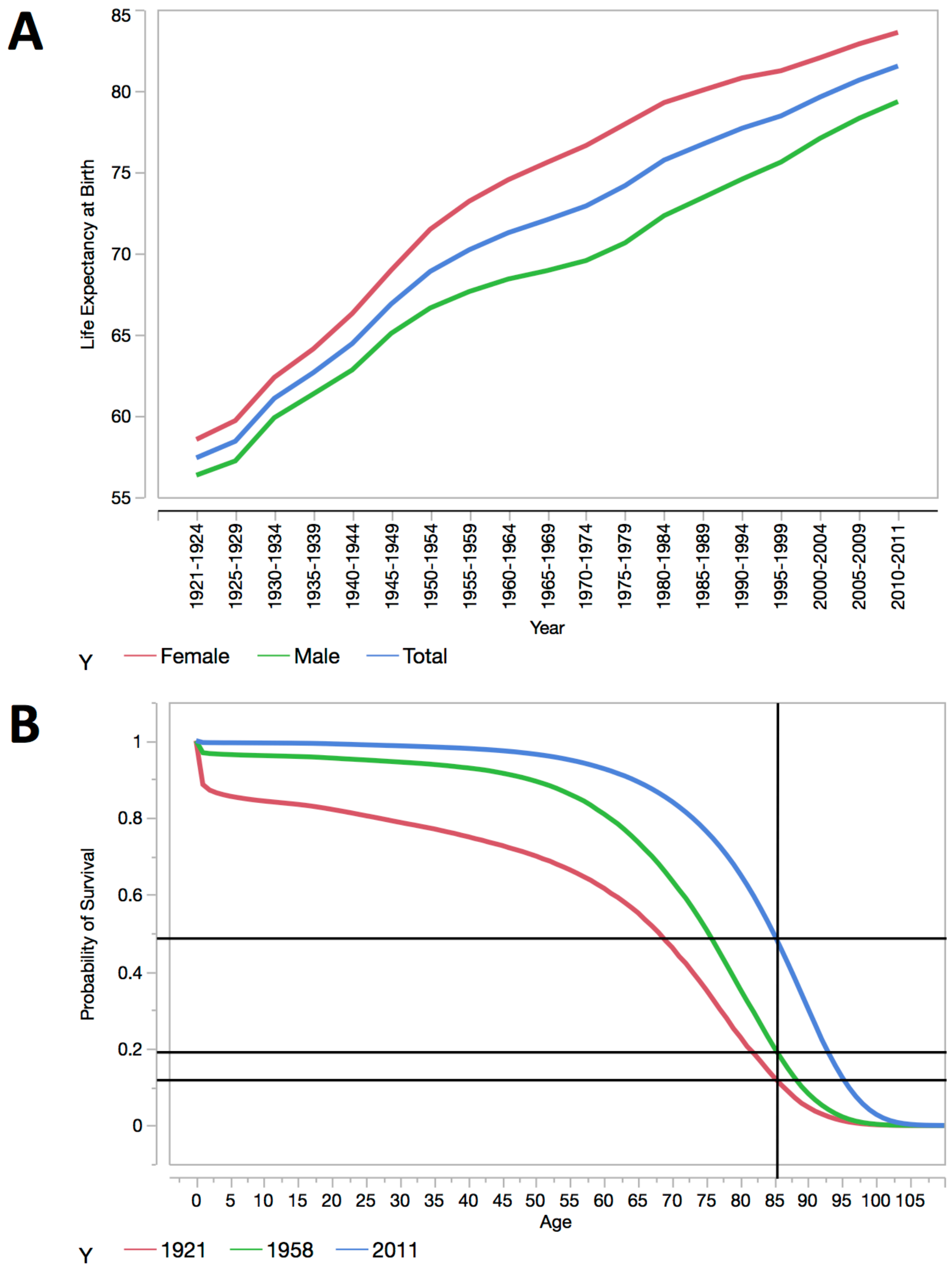

S1 Fig. Life expectancy in Canada. Data from the Human Mortality Database [15]. (A) Life expectancy at birth in Canada from 1921 to 2011. (B) Survival curve for Canada in 1921, 1958 and 2011. The Super-Seniors were born between 1901 and 1922 with a mean birth year of 1916; the controls were born between 1952 and 1964 with a mean birth year of 1958.
