# Supplementary material for: The Super-Seniors Study: Phenotypic characterization of a healthy 85+ population
Source: PLoS One. 2018 May 24;13(5):e0197578. doi: 10.1371/journal.pone.0197578 (PMC5967696; doi:10.1371/journal.pone.0197578)
Supplement: S2 Fig — (PDF) [file pone.0197578.s002.pdf]

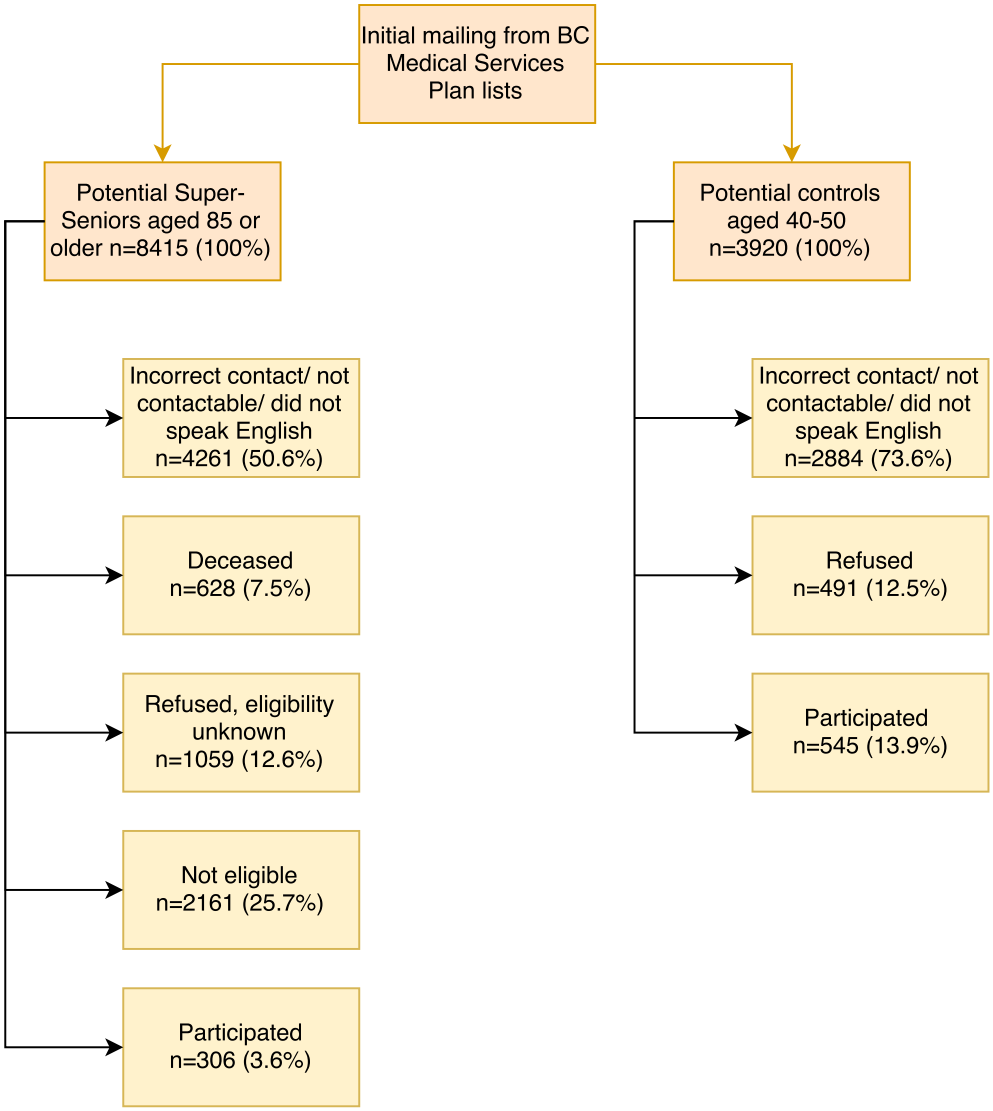

S2 Fig. Collection of Super-Seniors and controls from BC Medical Services Plan lists.

Consent rate for controls = 545 / (545+491) = 52.6%

Eligibility rate of contactable potential Super-Seniors = 306 / (306+2161) = 12.4%

How elite is the Super-Senior phenotype? 12.4% of seniors over age 85 who were contactable and interested were eligible. Taking into account that 28.5% of Canadians age 85 or older have dementia (Ebly *et al.*, *Neurology* 1994;44(9):1593-600, Prevalence and types of dementia in the very old: results from the Canadian Study of Health and Aging) and that such individuals are unlikely to be contactable and able to understand a phone interviewer, the eligibility rate of living individuals would be closer to 12.4% x (1-0.285) = 8.9%. Furthermore, only 9.0% of individuals born in 1916 lived to be 85 (Bell and Miller, 2005; Actuarial Study No. 120, Social Security Administration SSA Pub. No. 11-11536); interpolated from data for 1900 and 1950 birth cohorts. Therefore, the proportion of the 1916 birth cohort who went on to become Super-Seniors is approximately 8.9% x 9.0% = 0.80%.
