# Supplementary material for: The Super-Seniors Study: Phenotypic characterization of a healthy 85+ population
Source: PLoS One. 2018 May 24;13(5):e0197578. doi: 10.1371/journal.pone.0197578 (PMC5967696; doi:10.1371/journal.pone.0197578)
Supplement: S3 Fig — (PDF) [file pone.0197578.s003.pdf]

A. Age distribution of Super-Seniors

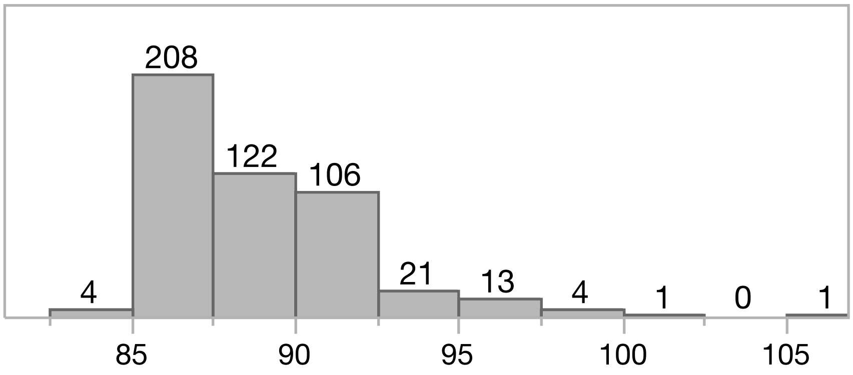

B. Age distribution of mid-life controls

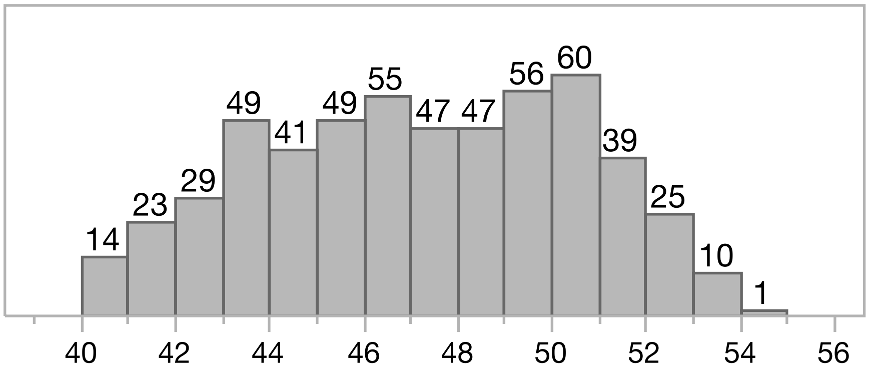

S3 Fig. Age distribution of participants in the Super-Seniors Study.
