# Supplementary material for: The Super-Seniors Study: Phenotypic characterization of a healthy 85+ population
Source: PLoS One. 2018 May 24;13(5):e0197578. doi: 10.1371/journal.pone.0197578 (PMC5967696; doi:10.1371/journal.pone.0197578)
Supplement: S1 Table — (PDF) [file pone.0197578.s004.pdf]

S1 Table. Distribution of ethnicity in Super-Seniors and controls.

	Super-Seniors			Controls
	Exclude	Borderline	Include	Include
First nations			1	
African				1
East Asian	2		18	57
European	56	16	444	416
Latin American			1	4
Middle Eastern	1		1	6
Mixed	1		3	24
South Asian	1		3	11
Unknown	2	1	9	26
Total	63	17	480	545
