# Supplementary material for: The Super-Seniors Study: Phenotypic characterization of a healthy 85+ population
Source: PLoS One. 2018 May 24;13(5):e0197578. doi: 10.1371/journal.pone.0197578 (PMC5967696; doi:10.1371/journal.pone.0197578)
Supplement: S2 Table — (PDF) [file pone.0197578.s005.pdf]

S2 Table. Contingency table of smoking status in Super-Seniors and controls.

		Current	Never	Quit	
**Super-Seniors**	Count	7	241	232	480
	Cell X^2	18.0	0.03	2.0	
**Control**	Count	58	268	219	545
	Cell X^2	15.9	0.03	1.8	
Total		66	509	450	1025
