# Supplementary material for: The Super-Seniors Study: Phenotypic characterization of a healthy 85+ population
Source: PLoS One. 2018 May 24;13(5):e0197578. doi: 10.1371/journal.pone.0197578 (PMC5967696; doi:10.1371/journal.pone.0197578)
Supplement: S3 Table — (PDF) [file pone.0197578.s006.pdf]

S3 Table. Contingency table of number of offspring in Super-Seniors and controls.

Offspring		0	1	2	3	4+	
**Super-Seniors**	Count<br>Cell X^2	49<br>19.3	63<br>0.2	140<br>2.5	107<br>3.4	121<br>31.5	480
**Control**	Count<br>Cell X^2	145<br>17.0	79<br>0.2	202<br>2.2	84<br>3.0	35<br>27.7	545
Total		194	142	342	191	156	1025
